# Supplementary material for: Head louse egg and nit remover—a modern “Quest for the Holy Grail”
Source: PeerJ. 2019 Apr 15;7:e6759. doi: 10.7717/peerj.6759 (PMC6472470; doi:10.7717/peerj.6759)
Supplement: Supplemental Information 1 [file peerj-07-6759-s001.docx]

Dataset for Figure 2

| Paranix Apres | | Ecrinal Poux | | KO Poux |  | Puressentiel | | KO Lentes | | Stubbon Egg Remover | | OTC Antipiojos | | Nit free mousse | | Dry Hair |
| --- | --- | --- | --- | --- | --- | --- | --- | --- | --- | --- | --- | --- | --- | --- | --- | --- |
| 150 |  | 38.3 |  | 199.1 |  | 351.1 |  | 32.4 |  | 11.8 |  | 48.1 |  | 42.9236 |  | 204 |
| 107.9 |  | 197.1 |  | 530.5 |  | 115.7 |  | 79.4 |  | 17.7 |  | 139.3 |  | 55.84235 |  | 223.6 |
| 22.6 |  | 44.1 |  | 105.9 |  | 32.4 |  | 103 |  | 17.7 |  | 52 |  | 172.9446 |  | 194.2 |
| 324.6 |  | 208.8 |  | 47.1 |  | 96.1 |  | 150 |  | 45.1 |  | 16.7 |  | 93.34841 |  | 180.4 |
| 117.7 |  | 79.4 |  | 220.7 |  | 155.9 |  | 51 |  | 33.3 |  | 71.6 |  | 65.42723 |  | 407 |
| 313.8 |  | 121.6 |  | 147.1 |  | 405 |  | 42.2 |  | 10.8 |  | 77.5 |  | 206.7 |  | 116.7 |
| 35.3 |  | 75.5 |  | 205.9 |  | 144.2 |  | 106.9 |  | 16.7 |  | 39.2 |  | 413.8168 |  | 154 |
| 77.5 |  | 344.2 |  | 127.5 |  | 128.5 |  | 125.5 |  | 16.7 |  | 78.5 |  | 243.7894 |  | 205.6 |
|  |  |  |  |  |  |  |  |  |  |  |  |  |  | 142.1063 |  |  |

Raw data used for construction of the box and whisker plot shown in Figure 2. The figures are measurements of Peak force read directly from the digital output screen of the slip-peel tester and converted to millinewtons.
